# Many Genes—One Disease? Genetics of Nephronophthisis (NPHP) and NPHP-Associated Disorders

**DOI:** 10.3389/fped.2017.00287

**Published:** 2018-01-05

**Authors:** Shalabh Srivastava, Elisa Molinari, Shreya Raman, John A. Sayer

**Affiliations:** ^1^Institute of Genetic Medicine, Newcastle University, Newcastle upon Tyne, United Kingdom; ^2^Renal Unit, City Hospitals Sunderland and South Tyneside NHS Foundation Trust, Sunderland, United Kingdom; ^3^Department of Histopathology, Newcastle upon Tyne Hospitals NHS Foundation Trust, Newcastle upon Tyne, United Kingdom; ^4^Renal Services, Newcastle upon Tyne Hospitals NHS Foundation Trust, Newcastle upon Tyne, United Kingdom

**Keywords:** ciliopathy, molecular genetics, nephronophthisis, cilia, centrosome, DNA damage, cyclic adenosine monophosphate, Joubert syndrome

## Abstract

Nephronophthisis (NPHP) is a renal ciliopathy and an autosomal recessive cause of cystic kidney disease, renal fibrosis, and end-stage renal failure, affecting children and young adults. Molecular genetic studies have identified more than 20 genes underlying this disorder, whose protein products are all related to cilia, centrosome, or mitotic spindle function. In around 15% of cases, there are additional features of a ciliopathy syndrome, including retinal defects, liver fibrosis, skeletal abnormalities, and brain developmental disorders. Alongside, gene identification has arisen molecular mechanistic insights into the disease pathogenesis. The genetic causes of NPHP are discussed in terms of how they help us to define treatable disease pathways including the cyclic adenosine monophosphate pathway, the mTOR pathway, Hedgehog signaling pathways, and DNA damage response pathways. While the underlying pathology of the many types of NPHP remains similar, the defined disease mechanisms are diverse, and a personalized medicine approach for therapy in NPHP patients is likely to be required.

## Introduction

Nephronophthisis (NPHP) is an autosomal recessive inherited kidney disease, which leads to end-stage renal disease (ESRD) typically within the first three decades of life (1). Traditionally, this disease was diagnosed using clinical and histological features. However, over recent years, many of the genetic causes underlying NPHP have been identified allowing both a precise molecular diagnosis to be made and some mechanistic insights into the underlying disease process. The known NPHP genes encode proteins that are almost all expressed in centrosomes and primary cilia. NPHP is therefore considered to be a ciliopathy disease (2), consistent with the fact that extrarenal manifestations, consistent will a ciliopathy syndrome, occur in around 20% of cases. Here we will review the clinical and histological features of the disease and its conventional classification before reviewing the underlying genetic causes and the ciliopathy syndromes associated with NPHP.

Based on the original histological descriptions, which included corticomedullary cysts, atrophy, and interstitial fibrosis, NPHP literally means disappearance or disintegration of nephrons (3). The clinical symptoms of NPHP, which reflect reduction in GFR and loss of distal tubular function (4), include polyuria, polydipsia, secondary enuresis, and growth retardation. Unfortunately, NPHP is associated with a progressive loss of kidney function and ESRD typically occurs before 30 years of age. Cases have historically been classified based on the age of onset of ESRD as infantile, juvenile, adolescent, and late onset. These are worth reviewing, although it is worth noting that a single genotype may present at a wide range of ages.

Juvenile NPHP is the classical form of NPHP and is characterized by polyuria and polydipsia symptoms and often anemia in patients within the first decade of life. Progressive loss of kidney function leads to ESRD at a median age of 13 years (5).

Kidneys affected by NPHP are grossly normal or have a shrunken appearance, typical of ESRD. There may be corticomedullary cysts that are up to 1.5 cm in size. Cysts often develop in later stages of the disease. The renal ultrasound scan appearances may often display a loss of corticomedullary differentiation.

**Figure 1:**
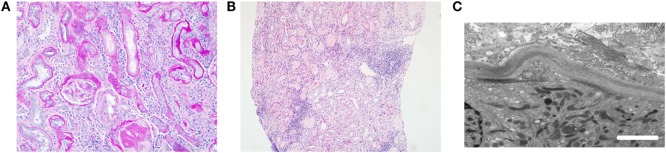
Typical histological features of nephronophthisis. **(A)** Light microscopy image. PAS stain demonstrates a globally sclerosed glomerulus and some periglomerular fibrosis. There is moderate interstitial fibrosis with chronic inflammation and thickening of tubular basement membranes. **(B)** Light microscopy image. H&E stain shows tubular atrophy with hyaline casts, moderate interstitial fibrosis, and patchy mononuclear inflammation. **(C)** Electron microscopy image. Tubular basement membrane demonstrates thickening and multilayering. Scale bar 2 µm.

Where renal biopsies have been performed in NPHP patients, distinct histological features have been reported. The histological changes can be divided into early or late stages of disease. In the early stages of the NPHP, there is interstitial fibrosis with sparse inflammation and lack of infiltration with neutrophils or monocytes. The tubules are tortuous and atrophic with segmented tubular basement membrane thickening (6). The distal tubules have focal diverticulum like protrusions. The glomeruli are usually normal but there may be periglomerular fibrosis that can extend into the glomerular tuft leading to focal or global collapse of the tuft and obsolescence of the glomeruli (7, 8). In later stages of the disease, the tubules may demonstrate basement membrane abnormalities with both atrophy and thickening. There often is cystic dilatation of the distal tubules, and the glomeruli may show collapse and severe periglomerular fibrosis (8, 9) (Figure 1). NPHP is not an immune mediated disease, and consequently there is no immune or complement deposition (6, 8). Electron microscopy may reveal tubular basement membrane duplication, thickening, and folding (6, 8). When examining clinical, pathological, and histological features of NPHP, it must be remembered that a separate disorder, known as medullary cystic kidney disease may share similar features. Medullary cystic kidney disease is an autosomal dominant condition, which is now classified under the term autosomal dominant tubulointerstitial kidney disease (ADTKD). Typical extrarenal manifestations include gout and anemia. A comparison of NPHP and ADTKD, alongside is given in Table 1 and has been discussed elsewhere (10).

**Table 1:** Comparison of nephronophthisis (NPHP) with autosomal dominant tubulointerstitial kidney disease (ADTKD).

Diagnosis	NPHP	ADTKD
Inheritance	Autosomal recessive	Autosomal dominant
Gene(s)	*NPHP* genes	*MUC1* *UMOD* *REN* *SEC61A1*
Extrarenal associations	Retinal degeneration Cerebellar vermis aplasia, gaze palsy, liver fibrosis, situs inversus, and skeletal defects	Gout
Radiological features	Small or normal-sized hyperechogenic kidneys and corticomedullary cysts (except infantile variant)	Small or normal sized hyperechogenic kidneys and corticomedullary cysts
Median age of end-stage renal disease	Usually under 30 years	16–80 years (*MUC1*) 30–50 years (*UMOD*)

Infantile NPHP is rare, but is noteworthy, due to its severe phenotype with ESRD typically occurring during the first year of life (7). There may be antenatal presentations with oligohydramnios and bilateral enlarged cystic kidneys. Infantile NPHP is usually caused by mutations in *INVS* (11) and *NPHP3* (12) but has been reported for other genetic forms of NPHP such as *NEK8* (13) and *CEP83* (14). The macroscopic and histological kidney phenotype is markedly different from other varieties of NPHP, with enlarged cystic kidneys, as opposed to micro and small corticomedullary cysts. Histologically, infantile NPHP lacks the tubular basement membrane changes seen in other NPHP phenotypes and may resemble autosomal recessive polycystic kidney disease. There may also be severe cardiac anomalies including situs inversus and ventricular septal defects (15).

The adolescent form of NPHP was originally described in a large Venezuelan pedigree (16). Biallelic mutations in *NPHP3* were found in this family, resulting in ESRD at a median age of 19 years (16). It is now known that *NPHP3* mutations may lead to a broad range of phenotypes including perinatal lethal Meckel–Gruber syndrome and infantile presentations. The term “adolescent NPHP” is thus somewhat arbitrary and merely extends the phenotypic spectrum from juvenile NPHP.

A number of case reports have highlighted the fact that NPHP may first present later in life. Georges et al. reported three (genetically unsolved) families with retinal dystrophy, NPHP on renal biopsy and slowly progressive renal failure and ESRD between the ages of 42 and 56 years (17). In another family with a homozygous *NPHP1* deletion (18) ESRD was reported in three patients between 27 and 43 years of age. These cases of NPHP extend the age of ESRD from birth to up to the sixth decade of life.

## An Approach to the Clinical Diagnosis of NPHP

Clinical recognition of NPHP is important, and the renal and extrarenal features of a ciliopathy syndrome (discussed below) may allow a clinical diagnosis to be made. NPHP occurs in isolation in around 80% of cases and is associated with a variety of other ciliopathy phenotypes in 20% of cases. A detailed review with specific emphasis on the family history and extrarenal features known to be associated with NPHP is therefore an essential prerequisite to an exact diagnosis. NPHP is characterized by a urinary concentrating defect early on in life that leads to polyuria and polydipsia. The onset of the disease may be easily missed, as there is typically no severe hypertension, minimal or no proteinuria, and a bland urine sediment. Clinical spectrums of disease are wide and widening. Besides extensive investigations of renal function, clinical phenotyping should also encompass a full neurological screening to assess for cerebellar signs and fundoscopy to assess for retinal degeneration. A formal ophthalmological examination is advised. The role of renal biopsy in diagnosing NPHP is contentious and should be limited to cases where a tissue diagnosis will serve to distinguish it from other differential diagnoses. In most cases, a histopathological diagnosis should be superseded by a molecular genetic diagnostic approach, because genetic screening allows for early diagnosis and prevents complications of renal biopsy. *NPHP1* mutations and deletions are the most frequent genetic cause of NPHP and may be screened for using standard PCR assays (19). Given the large numbers of other NPHP genes involved multiplex PCR (20), targeted exon capture or whole-exome sequencing approaches are recommended (21).

## Extrarenal Manifestations of NPHP

There are several important additional phenotypes that may be associated with NPHP (Table 2). These multisystem features are consistent with the fact that NPHP is a ciliopathy and may affect retina, brain, liver, and other tissues either by prenatal-onset dysplasia or by postnatal organ degeneration and fibrosis. Extrarenal manifestations are seen in ~20% of cases (22). In a recent study where 89 patients with NPHP mutations were analyzed, *NPHP1* mutations were the most common genetic cause and gave rise to typical renal presentations. These included increased echogenicity of the kidney and loss of corticomedullary differentiation, with cystic kidney disease presenting later in the disease course (median age 12.3 years) and ESRD at a median age of 12.8 years (23). Extrarenal manifestations of *NPHP1* mutations were seen more frequently than expected, with 8% presenting with liver symptoms, 19% having developmental delay and 7% epilepsy and seizures (23). The important syndromes associated with NPHP are briefly described below.

**Table 2:** Extrarenal manifestations of nephronophthisis (NPHP) and their associated syndromes.

Extrarenal manifestation associated with NPHP	Syndrome
Retinitis pigmentosa/retinal dystrophy	Senior–Løken syndrome
	Alström syndrome
	Arima syndrome
Oculomotor apraxia	Cogan syndrome
Nystagmus	Joubert syndrome and related disorders
Ocular coloboma	Joubert syndrome and related disorders
Posterior encephalocele	Meckel–Gruber syndrome
Abnormal respiratory pattern	Joubert syndrome and related disorders
Cerebellar vermis aplasia/hypoplasia	Joubert syndrome and related disorders
Liver fibrosis	Joubert syndrome and related disorders
	Meckel–Gruber syndrome
	Arima syndrome
Postaxial polydactyly	Bardet–Biedl syndrome
	Joubert syndrome and related disorders
Skeletal dysplasia	Ellis–van Creveld syndrome
	Sensenbrenner syndrome
	Jeune syndrome
	Mainzer–Saldino syndrome
Situs inversus/cardiac malformation	Infantile NPHP

### NPHP with Retinitis Pigmentosa (Senior–Løken Syndrome)

Retinal dysplasia and degeneration is seen in 10–15% of patients with NPHP and may lead to an early and severe visual loss resembling Leber congenital amaurosis (LCA) (24, 25). Later onset forms present initially with night blindness, which then progresses to visual loss.

### Cerebellar Vermis Aplasia/Hypoplasia with NPHP (Joubert Syndrome)

Joubert syndrome is a developmental disorder characterized by cerebellar vermis hypoplasia (26). Brain imaging (MRI) reveals the typical sign known as the “molar tooth sign.” Clinical features include hypotonia, cerebellar ataxia, neonatal tachypnea, and developmental delay. There may also be ocular coloboma, polydactyly, and hepatic fibrosis. NPHP is found in up to 30% of Joubert syndrome patients (27–29). Large cohorts of Joubert syndrome patients have been described, allowing some genotype/phenotype correlations to be made in the more frequent genetic causes. Mutations in *TMEM67* in Joubert syndrome are the most frequently associated with kidney disease, whereas mutations in *CEP290* were most likely to give retinal, renal, and brain phenotypes (28). In a recent cohort analysis of 97 patients with Joubert syndrome, renal phenotypes were detect in 30% of cases and was commonly associated with NPHP genes including *CEP290, TMEM67*, and *AHI1* (29). In this study, renal phenotypes in Joubert syndrome extended beyond classical NPHP and included an overlapping phenotype resembling autosomal recessive polycystic kidney disease and NPHP (mimicking infantile NPHP), unilateral multicystic dysplastic kidney and indeterminate cystic kidney disease phenotypes (29).

### Oculomotor Apraxia (OMA) Type Cogan

Oculomotor apraxia type Cogan is an eye movement disorder. It is characterized by abnormal horizontal eye movements that include nystagmus and difficulty with saccades (smooth visual pursuits) and has been associated with NPHP (2, 30). OMA may be a mild form of Joubert syndrome, as cerebellar vermis aplasia has been described in this condition (31).

### Perinatal Lethality (Meckel–Gruber Syndrome)

Meckel–Gruber syndrome is characterized by occipital encephalocele, polydactyly, bile ductal proliferation, and cystic kidney dysplasia. Typically, the condition is perinatally lethal. The syndrome is associated with severe biallelic mutations in NPHP genes that include *NPHP3, CEP290*, and *RPGRIP1L* (1, 32–35).

### Skeletal Defects [Jeune Syndrome (JS), Sensenbrenner Syndrome, and Saldino-Mainzer Syndrome]

Various skeletal defects have been reported in association with NPHP. These include cone-shaped epiphyses (16, 36), shortening of limbs, and ribs, scoliosis, polydactyly, brachydactyly, and craniosynostosis. Mutations are in genes encoding intraflagellar transport (IFT) proteins including TTC21B and WDR19 (37–41).

### Episodic Hyperpnea (Joubert Syndrome)

The original report of Joubert syndrome (42) described episodes of fast breathing followed by a period of apnea. This feature is demonstrable only when the patient is awake. Abnormal respiratory pattern is not a consistent feature of Joubert syndrome and the reported incidence varies (44–71%) (43).

### Anosmia As an Extrarenal Manifestation of Renal Ciliopathies

Several renal ciliopathy syndromes have been associated with anosmia, secondary to olfactory cilia defects. This has been studied in most detail in Bardet–Biedl syndrome (BBS) (44, 45) but has been reported in patients with LCA secondary to mutations in *CEP290* and in a murine model of *Cep290* (46). Such data also suggest a link between ciliary defects in the olfactory neurons and kidney disease. Indeed, proteins that mediate olfactory-like chemosensory signaling pathways were found expressed in the renal tissue (47), including adenylate cyclase III, which is localized to the primary cilium (48). These pathways may be vital in tubuloglomerular feedback and blood pressure control. There is a real need now to assess patients with renal ciliopathies/NPHP (and indeed corresponding murine models) for defects in smell and to determine a role for olfactory-like signaling within the kidney.

## Known Genetic Causes of NPHP

There are now more than 20 genes that if mutated may lead to NPHP (Table 3). It is worth reviewing these genetic causes as they all point toward some mechanistic insights into the pathogenesis of NPHP. Finding commonality among the genetic causes relies on a connection to the centrosome/basal body/primary cilium, although this may not be true for every genetic cause.

**Table 3:** Genetic classification of NPHP, its related disorders and the key insights obtained.

HGNC gene symbol	NPHP type	Disorders associated with mutations	Key insights	Reference
*NPHP1*	NPHP1	NPHP/SLSN/JBTS	Localizes to microtubular organizing center, cell–cell junction, and ciliary transition zone protein	(49, 50)
*INVS*	NPHP2	NPHP/SLSN (including infantile NPHP) situs inversus	Localizes to primary cilium (inversin compartment). Protein possesses nuclear localization signals suggesting role in nucleus. Role in Wnt signaling and left–right determination	(11)
*NPHP3*	NPHP3	NPHP/SLSN/MKS (including infantile NPHP)	NPHP3 interacts with ANKS6, INVS, and NEK8 in a ciliary protein complex. Murine model *Pcy* has a hypomorphic *Nphp3* allele and has been utilized in pharmacological studies	(16, 51)
*NPHP4*	NPHP4	NPHP/SLSN	Localizes to primary cilia and cell–cell junctions. Role in transition zone of cilia	(52)
*IQCB1*	NPHP5	SLSN/LCA	Localizes to connecting cilium of photoreceptor cells and to primary cilia of renal tubular cells	(53)
*CEP290*	NPHP6	JBTS/BBS/MKS/LCA/SLSN	Centrosomal protein localizes to mother and daughter centrioles and to transition zone. Protein contains a nuclear localization signal and localizes to nucleus	(33, 54, 55)
*GLIS2*	NPHP7	NPHP	Localizes to nucleus and primary cilia. Increase in apoptosis and fibrosis in murine model Glis2	(56)
*RPGRIP1L*	NPHP8	JBTS/MKS	Ciliary transition zone protein and facilitates vesicular docking of ciliary proteins	(1, 57)
*NEK8*	NPHP9	NPHP (including infantile NPHP)	Localizes to primary cilium (inversin compartment). Links cilia and cell cycle defects in NPHP	(13)
*SDCCAG8*	NPHP10	SLSN/BBS	Localizes to centrosomes and cell–cell junctions. Localized in nucleus and implicated in DNA damage repair pathways	(58, 59)
*TMEM67*	NPHP11	NPHP/MKS/JBTS/COACH syndrome	Localizes to transition zone and to plasma membrane. Required for ciliogenesis and centriole migration	(60–62)
*TTC21B*	NPHP12	NPHP/JBTS	Intraflagellar transport (IFT) protein involved in retrograde transport. Localizes to cilia.	(37)
*WDR19*	NPHP13	NPHP/JBTS	IFT protein, involved in retrograde transport and ciliogenesis	(38, 39)
*ZNF423*	NPHP14	JBTS	Nuclear and centrosomal protein and role in DNA repair signaling. Znf423−/− mice exhibit cerebellar defects	(63, 64)
CEP164	NPHP15	NPHP/SLSN/JBTS	Centrosomal protein and role in DNA repair signaling. Plays a critical role in G2/M checkpoint and nuclear division. Localizes to nuclear foci	(63)
ANKS6	NPHP16	NPHP	Functional module with inversin and nephrocystin-3	(65, 66)
IFT172	NPHP17	NPHP/Jeune/Mainzer–Saldino syndrome	IFT protein (IFT-B module), defects affect both anterograde and retrograde IFT	(67)
CEP83	NPHP18	NPHP (including infantile NPHP)	Distal appendage protein of centriole, required for membrane contact during ciliogenesisis	(14, 68)
DCDC2	NPHP19	NPHP/liver fibrosis	Ciliary and mitotic spindle protein, mutations disrupt Wnt signaling	(69)
MAPKBP1	NPHP20	NPHP	Mitotic spindle pole protein	(70)
AHI1	JBTS3	NPHP/JBTS	Localized to basal bodies and cell–cell junctions. Important for cerebellar development	(71–74)
CC2D2A		JBTS/COACH syndrome/MKS	Transition zone protein, regulates cilia-directed cargo vesicle docking	(75)
XPNPEP3	NPHPL1	NPHP	Mitochondrial defect linking ciliopathies with mitochondria and potential novel pathways of disease	(76)
ATXN10		NPHP/spinocerebellar ataxia	Interacts with IQCB1. Nuclear and cytoplasmic localization	(77)
SLC41A1		NPHP-like/primary ciliary dyskinesia	Renal magnesium transporter defect. Ciliary localization not confirmed	(78)

*BBS, Bardet–Biedl syndrome; COACH, cerebellar vermis hypo/aplasia, oligophrenia (mental retardation), ataxia, ocular coloboma, and hepatic fibrosis; JBTS, Joubert syndrome; LCA, Leber congenital amaurosis; MKS, Meckel syndrome; NPHP, nephronophthisis; SLNS, Senior–Løken syndrome*.

The most common genetic cause of NPHP is mutations in *NPHP1*, which account for around 20% of cases. The most common *NPHP1* gene defect is a large homozygous deletion affecting the whole gene (49, 79). Each of the remaining NPHP genes probably accounts for 1% or less of all cases of NPHP, meaning that around two-thirds of cases remain genetically unsolved (2). It is noteworthy that mutations in a single NPHP gene may give an extremely wide spectrum of clinical phenotypes that may include isolated NPHP, NPHP with additional features, such as Senior–Løken syndrome and Joubert syndrome and severe neonatal lethal forms, such as of Meckel–Gruber syndrome. Linkage studies and painstaking mapping approaches led to the identification of *NPHP1* in 1997 (49). Similar approaches for the next decade (sometimes combined with candidate gene screens) allowed the discovery of eight genes (at a rate of around one new gene per year). Since 2010, next-generation sequencing approaches have been utilized (80) allowing the detection of NPHP genes at a much faster rate.

### *NPHP1* 

*NPHP1* encodes nephrocystin-1 (alias nephrocystin). It was shown to interact with p130cas, tensin, filamin, and focal adhesion kinase 2, all molecules involved in cell–cell adhesion and cell signaling (81–83). In the primary cilium, nephrocystin-1 interacts with nephrocystin-4 and RPGRIP1L at the transition zone and links it to inversin (77).

### *INVS* 

INVS causes ESRD in the first 2 years of life and presents typically as an infantile form of NPHP as described earlier. The frequency of *INVS* mutations has been reported to be as high as 78% in the group of patients reaching ESRD before 2 years of age (12). The kidney size in INVS is often enlarged unlike most other forms of NPHP in which the kidneys are normal in size or shrunken (12). The distribution of cysts is corticomedullary and is more reminiscent of autosomal recessive polycystic kidney disease, given the kidneys can be massively enlarged.

Inversin, the gene product of *INVS* interacts with nephrocystin-1 and nephrocystin-3 and plays a vital role in intercellular adhesion (84). It localizes to the cilium and serves as a switch between the canonical and non-canonical Wnt pathway (85). Otto et al. established a link between cystogenesis and the primary cilia in humans disease during the study of this disease in 2003 (11) establishing this as a landmark paper in the study of NPHP and ciliopathies. Inversin is also plays a role in planar cell polarity (PCP) processes, discussed below. Loss of inversin leads to abnormal mitotic spindle orientation (86), which may drive cystogenesis.

### *NPHP3* 

Omran et al. first described mutations in *NPHP3* in a large Venezuelan family in 2000 (87). It is characterized by NPHP, situs inversus, and structural heart defects. Hoff et al. uncovered a link between nephrocystin-3, inversin, and NEK8 (65) in a report on the role of *ANKS6*, linking the above proteins at the proximal part of the primary cilium known as the inversin compartment. This may explain the overlap seen in the phenotype of patients with mutation in *INVS, NPHP3*, and *NEK8*.

### *NPHP4* 

*NPHP4* was identified by homozygosity mapping and genome wide linkage analysis by Mollet et al. in patients with NPHP who did not have mutations in the *NPHP1, 2*, and *3* genes. Nephrocystin-4 localizes to the primary cilia and cortical actin cytoskeleton in the polarized cells. In dividing cells, it localizes to the centrosomes. It has been shown to interact with p130 (Cas), tensin, and filamin (88).

### *IQCB1* 

Patients with *IQCB1* mutations are characterized by the presence of retinitis pigmentosa with NPHP (renal–retinal or Senior–Løken syndrome). In a study investigating the association of retinitis pigmentosa with NPHP, Otto et al. found a novel gene *IQCB1* that associates with retinitis pigmentosa GTPase regulator (RPGR) and calmodulin in the retinal connecting cilia, an analogous structure of the ciliary transition zone (53).

### *CEP290* 

Mutations in the *CEP290* gene underlie NPHP6 and are the leading cause of Joubert syndrome and related diseases, a cerebello–retinal–renal syndrome. The association of *CEP290* with NPHP was established in 2006 in a cohort of families with Joubert syndrome, Senior–Løken syndrome, and NPHP (54, 55). CEP290 was found to interact with the transcription factor ATF4, which is involved in cyclic adenosine monophosphate (cAMP) mediated cyst formation (54). *CEP290* mutations are the most common inherited cause of retinal degeneration (LCA). Mutations in *CEP290* may also cause BBS phenotypes (89).

TMEM67 (33, 90) and CC2D2A (91, 92) are both interacting partners of CEP290 and can cause severe ciliopathy phenotypes including Meckel–Gruber syndrome and Joubert syndrome. There is emerging evidence of the role of *CEP290* in ciliogenesis (93), cell signaling (94, 95), DNA damage response (DDR) (96), and consequently renal cystogenesis (97).

### *GLIS2* 

In 2007, Attanasio et al. reported a mutation in *GLIS2* as a novel cause for NPHP. The loss of this transcription factor leads to increased fibrosis and apoptosis (56). In a recent paper, *GLIS2* loss has been found to increase cell senescence. *Kif3a* null mice show increased cyst formation due to unrestrained proliferation, destabilization of p53 and increased DNA damage. This is partially rescued by ablation of *GLIS2* and pharmacological stabilization of p53 (98).

### *RPGRIP1L* 

Arts et al. identified mutations in *RPGRIP1L* as causative for Joubert syndrome in three families in 2007. This protein localizes to the basal body and interacts with NPHP4 (57).

### *NEK8* 

Otto et al. identified *NEK8* as the causative gene for Joubert syndrome after observing that the *jck* mouse harbors a mutation in the highly conserved RCC1 domain of Nek8. They performed a mutational analysis of a worldwide cohort of patients and established the pathogenic role of *NEK8* mutations in humans (13). More recently, *NEK8* loss was implicated in increased DNA damage in the pathogenesis of NPHP (99). This established one of the first associations between the role DDR and cystic kidney disease. Grampa et al. have recently described the association of *NEK8* with deregulation of the Hippo pathway and its role in severe syndromic renal cystic dysplasia (100). Al-Hamed et al. described a stillborn fetus with cystic kidneys, oligohydramnios, CVA and bilateral bowing of the femur secondary to *NEK8* mutation (101).

### *SDCCAG8* 

*SDCCAG8* was the first NPHP gene to be identified using next-generation sequencing approaches (58). Patients with mutations in this gene were diagnosed with Senior–Løken syndrome, but may also have features suggestive of BBS (102). The encoded protein SDCCAG8 localizes to centrioles and directly interacts with the ciliopathy-associated protein OFD1. A recently described murine model of SDCCAG8 has implicated elevated levels of DDR signaling as a potential mechanism of kidney disease (59).

### *TMEM67* 

Otto et al. screened a cohort of 62 patients with NPHP and liver fibrosis and found hypomorphic mutations in *TMEM67* in 8% of the patients (61). *TMEM67* has been implicated in the pathogenesis of Meckel–Gruber syndrome, Joubert syndrome, and COACH syndrome (cerebellar vermis hypo/aplasia, oligophrenia, congenital ataxia, coloboma and congenital hepatic fibrosis) (33, 34). Liver fibrosis is a frequent feature of *TMEM67* mutations, and any patient with NPHP along with liver involvement should have tests for mutations in *TMEM67*. In a cohort of 100 patients with Joubert syndrome, mutations in *TMEM67* were most frequently associated with kidney disease (28).

### *TTC21B* 

Davis et al. reported the association of *TTC21B* mutations with both isolated NPHP and JS (37). *TTC21B* encodes the retrograde IFT protein IFT139, which has been shown to regulate Hedgehog signaling (103).

### *WDR19* 

*WDR19* mutations have been reported in patients with ciliopathy syndromes including Sensenbrenner syndrome, Joubert syndrome, Senior–Løken syndrome, and isolated NPHP (38, 41, 104). *WDR19* encodes for IFT144, a protein that participates in retrograde IFT and is important for ciliogenesis.

### *ZNF423* 

*ZNF423* mutations have shown to cause Joubert syndrome with NPHP (63). The encoded protein ZNF423 is a nuclear protein which functions as a DNA-binding transcription factor and interacts with DDR protein PARP1 [poly (ADP-ribose) polymerase 1] and also CEP290 (63).

### *CEP164* 

Mutations in *CEP164* may cause NPHP and related ciliopathy syndromes including Senior–Løken syndrome (63). The CEP164 protein is a regulator of ciliogenesis and is essential for the formation of the distal appendage of the centriole (105). Loss of *CEP164* induces DNA damage (63).

### *ANKS6* 

*ANKS6* mutations lead to NPHP. ANKS6 localizes to the inversin compartment and links the NPHP proteins NPHP2, NPHP3, and NPHP9 to NEK8. This functional role of ANKS6 in an NPHP module may explain the phenotypic overlap that includes abnormalities in heart and liver, seen in the patients carrying individual mutations in these genes (65, 66).

### *IFT172* 

Intraflagellar transport is vital in maintaining the cilium and in executing its functions. IFT-A module has six components, and mutations in genes encoding these proteins have all been associated with ciliopathy diseases. IFT-B has 14 components. Halbritter et al. established the first link between IFT-B component IFT172 and skeletal ciliopathies. Some patients in this cohort had NPHP (67). Mutations in IFT172 may also cause BBS syndrome (106).

### *CEP83* 

*CEP83* mutations have recently been described to cause infantile NPHP (14). *CEP83* encodes a centriolar distal appendage protein, CEP83. In the seven families so far described, the NPHP phenotype was early-onset (juvenile), and in some was also associated with hydrocephalus and learning difficulties (14).

### *DCDC2* 

Schueler et al. reported a novel mutation in the gene *DCDC2*, in patients presenting with an NPHP and hepatic fibrosis phenotype (69). DCDC2 localizes to the ciliary axoneme and the mitotic spindles. *DCDC2* knockdown inhibits ciliogenesis. It interacts with DVL, and *DCDC2* knockdown leads to defects in Wnt signaling and may contribute to the liver fibrosis (69).

### *MAPKBP1* 

Macia et al. have recently described a novel gene, *MAPKBP1*, in five families with eight individuals presenting with juvenile or late-onset NPHP with massive fibrosis. This gene encodes MAPKBP1, a scaffolding protein for JNK signaling. Interestingly, this protein does not localize to the primary cilium instead it localizes to the mitotic spindle pole. The authors also report increased DDR signaling in murine fibroblasts upon knockdown of *Mapkbp1* (70).

### *AHI1* 

Mutations in AHI1 were initially described in patients with Joubert syndrome and no kidney involvement (71, 72). However, AHI1 mutations may cause NPHP phenotypes (73) and may cause multicystic dysplastic kidneys also (29). AHI1 is localized to the basal body and cell–cell junctions (74).

### *CC2D2A* 

Mutations in *CC2D2A* have been reported to cause Joubert syndrome with and without cystic kidney disease (92). CC2D2A is localized to the basal body and colocalizes with CEP290 (92). Mutations in *CC2D2A* may also cause antenatal cystic kidney disease phenotypes and severe brain phenotypes (typical of Meckel–Gruber syndrome) leading to fetal death (101).

## Evidence of Oligogenicity and Triallelism in NPHP

Alongside the novel findings relating to gene discovery in NPHP has been the continued theme of wide phenotypic variability, especially in extrarenal manifestations. The type of mutation may influence the phenotype in certain circumstances. Examples include *CC2D2A* (107, 108) and *TMEM67* (109) where two truncating mutations tend to lead to more severe phenotypes than missense mutations. With the now frequent sequencing of NPHP cohorts (110, 111) and the use of high-throughput genetic sequencing platforms (112), a few findings of oligogenicity and triallelism within NPHP have been reported; however, these are controversial as these findings are anecdotal. As an example, a heterozygous *AHI1* mutation when inherited with biallelic *NPHP1* mutations seems to lead to a more severe brain phenotype (111). Thus, a concept of mutation burden seems relevant to NPHP, and like BBS (113) it will be important that these variants are reported and that they are assessed in terms of their pathogenicity. Interestingly, *NPHP1* mutations and copy number variants, as well as causing NPHP and Joubert syndrome, may also contribute to the mutational burden of BBS (114, 115). More recent next-generation sequencing data studying Joubert syndrome suggest that rare disease variants are frequently found in addition to the causal biallelic variants. Typically, over one-third of affected individuals carry rare disease variants in addition to the causal mutations but importantly they did not correlate with disease severity (116). This study also found no evidence or support for triallelism. Discordant phenotypes between affected siblings were observed in 60% of subjects who shared causal mutations, suggesting that modifier alleles are important but elusive (116). Therefore, using third alleles and other NPHP gene variants of uncertain significance to determine additional phenotypes and disease severity and inform genetic counseling is not presently advised, or should only be done with the utmost care.

## Pathogenesis of NPHP

There are various theories behind the pathogenesis of the NPHP disease process. The very early hypotheses were based entirely on the histopathological description of the disease and led to the widespread belief that this disease was caused by some unknown nephrotoxic agent or an enzyme defect (8). The frequent finding of tubular basement membrane thickening led to a basement membrane hypothesis for the pathogenesis of NPHP. It was observed that nephrocystin-1, the protein product of *NPHP1* had a high degree of sequence conservation with CRK (a focal adhesion protein) (117), contained an SH3 domain and interacted with other proteins including p130Cas and ACK1 (49, 118). Nephrocystin-1 was shown to localize to adherens junctions and focal adhesions. This supported a hypothesis that nephrocystin-1 has an important role in the maintenance of the tubular epithelium and that abnormal cell–cell and cell–matrix interactions were the underlying defect in NPHP. Many years later, the debate of the initial pathogenic defect in NPHP continues, with the focus on NPHP as a ciliopathy (119). This hypothesis is strongly supported by multiple gene discoveries in NPHP with nearly all the affected genes coding for the components of the cilia, basal body or centrosome. Defects in primary cilia associated with cystic kidney disease were initially noted in *Ift88* mutant mice (120). This link between NPHP and cilia was confirmed in human disease established after the discovery that *INVS* mutations cause infantile NPHP and that the encoded protein inversin interacts with nephrocystin-1 and β-tubulin, colocalizing with them to the primary cilia of renal tubular cells (11). There is now almost universal agreement that the primary cilia are at the center of the disease process especially in terms of cystogenesis although it should not be forgotten that nephrocystins may have multiple subcellular localizations (54) and may play different roles in different tissues (1). There is an interesting overlap with the localization and function of NPHP genes and other “cystogenes” such as *PKD1, PKD2*, and the many other inherited causes of cystic kidney disease. The functional role of primary cilia in the human nephron is not fully understood. It was initially thought that the encoded proteins from *PKD1* and *PKD2*, namely, polycystin-1 and polycystin-2, were able to sense luminal flow of urine, and ciliary deflection stimulated calcium entry into the cell *via* the polycystin proteins leading to downstream signaling cascades (121). However, more recent studies have challenged this hypothesis (122, 123).

## A Comparison of NPHP and ADPKD Pathophysiology

A detailed discussion of the underlying pathophysiology of ADPKD has been recently published and is beyond the scope of this review (124). It is worth highlighting, however, that disease pathways in ADPKD involve cAMP, ciliary dysfunction, PCP and centrosome number as well as many others (124). Thus, these mechanisms of disease are shared with those of NPHP. Indeed, the development of tolvaptan, a vasopressin V2 receptor (V2R) antagonist for the use in patients with ADPKD was pioneered in murine models of NPHP (51). However, fluid secretion and proliferation seems less prominent in NPHP, while fibrosis and scarring are more prominent pathological features. The pairing of potential disease mechanisms in NPHP with targeted therapeutics will hopefully allow better treatments for NPHP in the near future (125).

## Other Key Molecular Pathways Implicated in NPHP

### Planar Cell Polarity

Planar cell polarity is an evolutionary conserved mechanism by which cells maintain their orientation in a plane perpendicular to the apical-basal polarity of a cell layer. This is achieved by the correct alignment and orientation of cell division, orchestrated by the mitotic spindle and centrosomes. The maintenance of tubular diameter is dependent upon PCP signaling, and when this is defective, tubular dilatation rather than elongation is thought to contribute to cystogenesis (126, 127). Non-canonical Wnt signaling is vital for these signaling events and mutations in *INVS* are thought to lead to defective regulation of this pathway (84, 85). Mutations in *DCDC2*, leading to NPHP type 19 have also been implicated in this pathway, lending weight to this mechanism of cytogenesis and NPHP. Loss of Dcdc2 in IMCD3 cells led to an activation of Wnt signaling, leading to a loss of cilia, which was amenable to treatment with Wnt inhibitor treatment (69). Other connections to the Wnt pathway in NPHP includes CEP164 which interacts with disheveled protein 3 (DVL3) (63). The disheveled protein is a key component of the Wnt pathway, and part of the switch between canonical and non-canonical Wnt signaling. Defective Wnt signaling has also been demonstrated in murine models of Joubert syndrome. *Ahi1* mutant mice showed defect in cerebellar midline fusion in sites of reduced Wnt activity (128) while renal tissues from the same mice demonstrated abnormal Wnt signaling in late stages of NPHP (129). A murine gene trap model of *Cep290* similarly showed Wnt pathway changes (reduced Tcf1 protein) only at later stages of the disease (murine kidney tissue aged 1 year) implicating this pathway in renal fibrosis (95). The relationship between Wnt signaling and cystic kidney disease has been recently reviewed (130).

### cAMP Signaling

A huge amount of data have demonstrated the key role of elevated cAMP in mural epithelial cell proliferation and fluid secretion, which are the main drivers of cyst formation in polycystic kidney disease (131). However, some lines of evidence suggest that high cAMP levels are also implicated in junctional and polarity defects in NPHP. Levels of cAMP were found to be elevated in *Nphp3-, Nphp6-*, and *Nphp8-*stable knockdown mIMCD3 lines. When these cells were examined in a 3D spheroid culture system, they formed abnormal spheroids with no lumen and/or misaligned nuclei. Treatment with octreotide, an inhibitor of cAMP production, could rescue these structural abnormalities, linking high cAMP levels to cell polarity defects (132). Furthermore, it was shown that treatment with the cAMP analog 8-bromo-cAMP resulted in a dose-dependent loss of SDCCAG8 (NPHP10) protein in cell–cell junctions of the renal epithelial cell line MDCK-II, highlighting the potential role of high cAMP in perturbation of tissue architecture (58). Importantly, elevated cAMP levels and expression levels of the cAMP-dependent gene *Aquaporin-2* were found in the renal tissue of *Pcy* mice that carry a missense mutation in *Nphp3* (51).

### mTOR Pathway

Increased mTOR (mechanistic target of rapamycin) activity was found in cystic kidney (133, 134) and in particular in cyst-lining epithelium (135) of several NPHP mouse models. mTOR is an atypical serine/threonine kinase that, by integrating a variety of signals from nutrients and growth factors, regulates cell growth and proliferation.

The detailed subcellular localization of mTOR pathway components is not clear but it has been showed that primary cilium is important for the regulation of mTOR pathway (136, 137). It has been proposed that the flow-dependent bending of primary cilium represents a mechanosensory signal that controls cell size through the regulation of mTOR activity (137). Consequently, cilia abnormalities may ultimately result in cell growth deregulation, which could be potentially critical for the tubular geometry of the kidney.

## The Role of Cilia in Sonic Hedgehog Signaling and Cell Cycle

The Hedgehog (Hh) signaling pathway is a key developmental pathway and was first discovered in *Drosophila* (138). There are three mammalian Hh homologs, Desert, Indian, and Sonic. The sonic Hh (Shh) pathway is essential for development (139), patterning, organogenesis, and cell signaling (140). It acts as a morphogen and a mitogen and dysregulation of the pathway can lead to severe developmental defects and can give rise to various cancers (141). Shh signaling is intimately related to the primary cilium (141). The receptor Patched (Ptch1) is 12-pass transmembrane protein localized to the primary cilium. It has an inhibitory effect on the translocation of Smoothened (Smo, a G-protein-coupled-like receptor). The secreted ligand Shh binds to Ptch1 and triggers internalization of Ptch1 into endocytic vesicles. This allows the translocation of Smo into the primary cilium and its stepwise activation (142). Downstream effector Glioma proteins (Gli2,3) remain in a neutral state under the effect of suppressor of fused and by sequential phosphorylation by protein kinase A, glycogen synthase kinase 3β, and casein kinase 1, they undergo proteolytic conversion to their repressor form. Smo, when enriched in the primary cilium and activated, promote the conversion of Gli repressor (Gli3r) forms into full-length activator forms. The Gli activators (Gli3a) induce the expression of Hh target genes *cyclin D1, Gli1, Gli2, N-myc*, and *Ptch1*. An intact Hh pathway is important for ciliogenesis. The evidence implicating the defects of the Hh signaling in NPHP, renal development and cystogenesis is evolving (77, 103). Loss of the transcription factor Glis2 (Gli-similar zinc finger protein) causes NPHP type 7 (56), Shh knockout mouse embryos showed either renal agenesis or cystic dysplasia (143) and upregulated Indian Hh has been implicated in cystogenesis (144). A subset of BBS proteins has been shown to modulate Shh signaling and interact with IFT proteins (145). More recently, Hh signaling has been shown to be dysregulated in models of cystic kidney disease including *Thm1, Pkd1, jck* (103) and *Cep290* (95).

## The Ciliary Transition Zone and Links to NPHP

Between the basal body and the ciliary axoneme lies the transition zone, a physical barrier between the ciliary membrane and the apical plasma membrane of the cell. Several genes causing NPHP encode transition zone components, including *NPHP1, RPGRIP1L, NPHP4*, and *CEP290*. The transition zone controls the protein entry into and exit from the primary cilium and the composition of the ciliary membrane, which directly impacts ciliary signaling pathways such as Hedgehog signaling. The hedgehog signaling molecule Smoothened has been shown to accumulate in discrete clusters in the transition zone, and RPGRIP1L mutations disrupted this localization, leading to disrupted signaling (146). These data support the hypothesis of the transition zone as a gatekeeper of the cilium and that defects can account for phenotypes such as NPHP.

## DDR Pathways and NPHP

The DDR signaling pathway allows the cell to detect DNA damage, apply an arrest in cell cycle and promote repair of the DNA. Repair of double stranded DNA breaks is particularly important for the maintenance of chromosome integrity. The DNA damage pathway ensures that damaged cells do not progress through S phase and into mitosis before repair is complete. Recently, several of the proteins implicated in NPHP including NEK8 (99), CEP164 (147), ZNF423 (63), SDCCAG8 (58, 59), and CEP290 (96) have been implicated in this pathway, suggesting a nuclear (non-ciliary) role. Following DNA damage, ZNF423, CEP164, and SDCCAG8 proteins have been shown to colocalize to nuclear foci positive for TIP60, a marker of sites of DNA damage and knockdown of CEP164 or ZNF423 causes increased sensitivity to DNA damaging agents (63). These observations provided a hypothesis that may explain why some NPHP genes with null mutations (such as *NPHP3, CEP290*, and *RPGRIP1L*) present as severe congenital-onset dysplasia and malformation in multiple organs including the kidney, brain and eye while hypomorphic mutations in the same genes produce milder phenotypes, which include late-onset degeneration and fibrosis leading to NPHP in the kidney and retinal degeneration in the eye. During periods of high proliferation and replication stress such as morphogenesis, DDR signaling is essential, and defects may lead to tissue dysplasia. By contrast, during maintenance of tissues in postnatal life, low replication stress would be expected, and defects would produce a degenerative phenotype. This hypothesis may go some way to explain the organ specific phenotypes seen in Joubert syndrome and other syndromes associated with NPHP (63). DDR defects and replication stress may also be an explanation for the fibrosis seen in association with NPHP and represents drugable target for the disease, which may be independent from and more reversible than cystogenesis (96, 148).

## An Integration of Signaling Pathways in the Development of NPHP

It remains clear that given the genetic heterogeneity of NPHP and the numerous mechanistic pathways discussed that there is not one unifying pathology leading toward NPHP. The renal histology of NPHP points to a common endpoint of tubular damage and fibrosis, which may have multiple triggers. With each new gene discovery paper, there seems to be better clarity toward molecular diagnosis but more confusion regarding the signaling pathways underlying disease.

The clear themes concerning NPHP are that this disease is a manifestation of a renal ciliopathy with almost all NPHP-associated genes encoding gene products known to localize to primary cilia and regulate ciliary function and structure (149), with both the Hedgehog and Wnt signaling pathways implicated downstream from abnormal ciliary signaling. The function of the ciliary transition zone as a gatekeeper for ciliary protein entry and exit is clearly fundamental to ciliary signaling processes. Protein interaction studies of NPHP proteins now allow the proteins to be grouped into four distinct modules. These are the NPHP1–4–8 (NPHP1, NPHP4, and RPGRIP1L) module, the NPHP2–3–9-ANKS6 (INVS, NPHP3, NEK8, and ANKS6) module, the NPHP5–6 (IQCB1 and CEP290) module and the MKS module (MKS1, CC2D2A, and TCTN2). This points to the fact that each NPHP protein has a distinct localization and function within the centrosome/transition zone/cilium.

However, there is also growing evidence for a nuclear/DDR function of some NPHP-associated proteins, which may be important in disease initiation and progression. Whether this is independent of roles in the primary cilium is not known. It is possible that loss of ciliary function may be a downstream effect of nuclear events affecting cell cycle progression as a result of replication stress (148). The intimate relationship between ciliogenesis and DDR has recently been discussed (150). Centrosomal proteins including NEK8 and CEP290 that are mutated in ciliopathy disorders and are known to have functional roles in DDR are discussed in detail. Overall, it seems likely, given the body of evidence concerning cilia and cystic kidney disease that ciliary dysfunction is a relatively specific subcellular phenotype and final common pathway leading to NPHP, but other pathways may feed into this and be interrelated.

## Treatment of NPHP

Nephronophthisis is incurable at present, but a range of potential therapeutic interventions has arisen from several lines of investigation into the pathogenesis of NPHP. Elevated renal cAMP levels were found associated with the cystic phenotype of NPHP and the modulation of cAMP production has been extensively explored as a potential strategy in the treatment of cystic kidney disease (51, 151–154). V2R antagonists are able to slow the rate of cAMP production by inhibiting V2R that, by coupling with G proteins, regulates the activity of adenylate cyclase and mediates urine concentration. Indeed, V2R antagonists OPC31260 and tolvaptan were shown to be effective in reducing renal accumulation of cAMP and rescuing the cystic kidney phenotype in *Pcy* mice (a model of NPHP3) (16, 51, 153). The use of tolvaptan has now moved successfully from preclinical models, through clinical trials (155) and into clinical practice (156). Its use in childhood ADPKD is currently being investigated in clinical trials.

As discussed, several lines of evidence support a direct link between DNA damage and the loss of NPHP proteins. In particular, both the NPHP type nine associated protein NEK8 and Cep290 were shown to be important regulators of DNA damage as their loss leads to increased sensitivity to replication stress and increased levels of CDKs (96, 99). Interestingly, CDK inhibition is able to suppress the DNA damage caused by loss of NEK8 or Cep290, therefore providing a rationale for CDK inhibition as a potential strategy in the treatment of NPHP (96, 99). Indeed, the CDK inhibitor roscovitine and its analog S-CR8 significantly halted the progression of cystic phenotype and attenuated loss of kidney function in *jck* mice (carrying a mutation in *Nek8*) (99, 157). Furthermore, roscovitine was able to ameliorate the ciliary phenotype of renal epithelial cells derived from a patient with NPHP secondary to a mutation in *CEP290*. Interestingly, it was shown that the treatment with purmorphamine, an agonist of the Shh pathway, is not only as effective as roscovitine in rescuing the ciliary defect, but is also able to decrease CDK5 protein levels in patient cells, suggesting a possible convergence of these signaling pathways (158).

Given the pivotal role played by the primary cilium in the context of Hh pathway, a manipulation of Hh signaling appears as an appealing strategy in the treatment of ciliopathies such as NPHP. It has been shown that genetic deletion of *Gli2* can ameliorate the cystic kidney phenotype in an orthologous mouse model of TTC21B (56), while Hh agonism mediated by purmorphamine treatment is able to rescue the architectural defect displayed by 3D cultures of CEP290 renal epithelial cells (95).

Hyperactivation of mTOR (mechanistic target of rapamycin) pathway was found to be associated with cystic kidney disease and rapamycin has proven to be effective in several rodent [Han:SPRD rat (134, 159), LPK rat (135), *Pcy* mouse (133), and zebrafish models (*invs, iqcb1*, and *cep290* morphant) of NPHP (160)].

The zebrafish models of NPHP are proving also to be extremely useful for high-throughput drug screens to determine their effect on kidney development (161).

There is hope therefore that these and other animal models of NPHP will provide valuable insights for future personalized medicine treatments of NPHP in affected patients (162). However, despite the great number of promising interventions that has arisen from preclinical studies, no clinical trials have yet been conducted to test their therapeutic potential in NPHP patients, most of whom eligible for treatment would be less than 18 years of age (125).

To date, options for the treatment of NPHP remain supportive. Control of blood pressure is a priority in children and young adults affected. Management of complications arising from progressive renal failure such as anemia, symptoms of uremia and fluid overload are important alongside preparation for future renal replacement therapy. This disease does not recur in a transplant and renal transplantation remains the ideal mode of renal replacement therapy.

## Conclusion

The clinical and pathological diagnosis of NPHP is important, given its progression to ESRD and its associated extrarenal manifestations. Molecular genetic investigations allows a diagnosis in around one-third of cases and can give insights into the associated disease features, the underlying mechanisms and hopefully pave the way for individualized treatments for the underlying kidney disease. As this review demonstrates, it is true that many genes cause NPHP and while most of the identified molecular causes implicate the primary cilium in the pathogenesis of NPHP, it has also become apparent that there are important differences in the underlying pathophysiology. The traditional descriptions of NPHP of infantile, juvenile, and adolescent may now seem dated; however, they highlight the fact that different genetic forms of the disease disrupt the kidney by different mechanisms, demanding a precision medicine approach to the diagnosis, understanding and treatment of NPHP and its associated syndromes.

## Author Contributions

JS conceived, drafted, and wrote the manuscript. SS and EM drafted and revised the manuscript. SR drafted the manuscript and provided figures.

## Conflict of Interest Statement

The authors declare that the research was conducted in the absence of any commercial or financial relationships that could be construed as a potential conflict of interest. The reviewer KH and handling Editor declared their shared affiliation.
